# Downregulation of miR-574-5p inhibits HK-2 cell viability and predicts the onset of acute kidney injury in sepsis patients

**DOI:** 10.1080/0886022X.2021.1939051

**Published:** 2021-06-16

**Authors:** Shanshan Liu, Lishu Zhao, Li Zhang, Lujun Qiao, Shufang Gao

**Affiliations:** a Emergent Intensive Care Unit, Shengli Oilfield Central Hospital, Dongying, China; b Department of Critical Care Medicine, Shengli Oilfield Central Hospital, Dongying, China

**Keywords:** Acute kidney injury, sepsis, miR-574-5p, human kidney tubular epithelial cell line, diagnostic

## Abstract

**Background:**

Increased levels of microRNA-574-5p (miR-574-5p) have been found to be associated with increased survival of septic patients, indicating the potential role of miR-574-5p in protecting against septic progression and complications. Acute kidney injury (AKI) is one of the most common and serious complications of sepsis. Therefore, the aim of this study was to test these hypotheses: (1) in a renal cell culture line (HK-2), upregulated expression of miR-574-5p increases, and downregulated expression of miR-574-5p decreases cell viability, and (2) serum levels of miR-574-5p from patients with sepsis and AKI are lower than those of patients with sepsis but no AKI.

**Methods:**

The expression of miR-574-5p was regulated by cell transfection in HK-2 cells, and HK-2 cell viability was measured using the Cell Counting Kit-8. Serum miR-574-5p expression was analyzed using qRT-PCR. The predictive value of miR-574-5p for AKI onset was evaluated using the receiver operating characteristic curve and logistic regression analysis.

**Results:**

The overexpression of miR-574-5p promoted HK-2 cell viability. Fifty-eight sepsis patients developed AKI, who had significantly lower miR-574-5p expression. miR-574-5p expression was decreased with AKI stage increase and correlated with kidney injury biomarker and had relatively high accuracy to predict AKI occurrence from sepsis patients.

**Conclusion:**

Overexpression of miR-574-5p in cultured HK-2 cells increases cell viability and knocked-down expression of miR-574-5p decreases cell viability. Consistently, septic patients with AKI were found to have less upregulation of miR-574-5p expression compared to septic patients without AKI. Thus, serum miR-574-5p may provide a novel biomarker for septic AKI.

## Introduction

Sepsis refers to systemic inflammatory response syndrome (SIRS) caused by infection [1]. The incidence of sepsis is high, with more than 18 million severe cases of sepsis worldwide and 750 000 cases of sepsis in the United States each year, and this number is increasing at a rate of 1.5–8.0% per year [2]. Sepsis can be caused by infection at any site. Clinically, sepsis-induced pneumonia, peritonitis, and cholangitis are common, however acute kidney injury (AKI) is one of the most common complications of sepsis [3]. AKI is a syndrome characterized by rapid loss of renal excretion function, and the main pathological mechanism of which is severe damage to kidney tissues and cells [4]. There are some molecular markers associated with renal injury, such as serum creatinine (Scr), cystatin C (Cys-C) and kidney injury molecule-1 (KIM-1) [5], but the accuracy of these molecular markers for the diagnosis of AKI is still limited.

MicroRNAs (miRNA) are an endogenous non-coding small RNA with a length of about 22 nt [6]. An increasing number of studies have shown that abnormal expression levels of miRNA plays an important role in the diagnosis, treatment and prognosis of a variety of diseases [7,8]. For example, Zhu et al. demonstrated that miR-214 can inhibit apoptosis by targeting Dkk3 and activating Wnt/catenin signaling pathway, which provides a possible target for AKI treatment [9]. Previous studies have shown that miR-574-5p is significantly associated with the survival of sepsis patients, the expression levels of miR-574-5p were higher in sepsis survivors compare with nonsurvivors [10]. In addition, miR-574-5p plays an important role in regulating cell viability. However, it is not clear whether miR-574-5p has an effect on renal epithelial cells viability and is associated with sepsis-induced AKI.

Thus, the purpose of this study was to test the hypotheses that (1) upregulated miR-574-5p expression in cultured renal cells increases cell viability, and (2) upregulation of serum miR-574-5p levels by sepsis is less robust if septic AKI occurs. In addition, the correlation of miR-574-5p with kidney injury biomarkers and the clinical significance of miR-574-5p in the prediction of AKI occurrence in sepsis patients were also evaluated. This study is expected to find new biomarkers for sepsis-induced AKI and ways to interfere with the progression of AKI.

## Method

### Cell culture and transfection

The human kidney tubular epithelial cell line (HK-2) was purchased from the Cell Bank of the Chinese Academy of Sciences (Shanghai, China). HK-2 cells were culture using Dulbecco’s Modified Eagle’s Medium/Nutrient Mixture F-12 (1:1, Gibco, CA, USA) supplemented with 10% fetal bovine serum (FBS; Gibco) in a cell incubator with 5% CO_2_ at 37 °C. To regulate the expression of miR-574-5p in HK-2 cells, the miR-574-5p mimic, miR-574-5p inhibitor, mimic negative control (NC) and inhibitor NC were synthesized by GenePharma (Shanghai, China), and were separately transfected into HK-2 cells using Lipofectamine 3000 (Invitrogen, CA, USA) following the manufactures’ protocols. After 48 h of transfection, the cells were used for further analyses. The transfection sequence was as follows (from 5′ to 3′): miR-574-5p mimics, UGAGUGUGUGUGUGUGAGUGUGU; miR-574-5p inhibitor, ACACACUCACACACACACACUCA; mimics NC, UUCUCCGAACGUGUCACGU; inhibitor NC, CAGUACUUUUGUGUAGUACAA.

### Patients and sample collection

This study analyzed 155 patients with sepsis in Shengli Oilfield Central Hospital from 2017 to 2019, diagnosis of sepsis was performed based on the diagnosis criteria from the International Sepsis Definition Conference [11]. Nineteen patients were excluded from the 155 patients with the following exclusion criteria: (i) two patients died within 72 h after admission; (ii) one patient younger than 18 years of age; (iii) two patients received nephrotoxin within 4 weeks before admission; (iv) four cases had immunodeficiency, severe hepatitis or coagulation disorder; (v) five patients had AKI before sampling; (vi) five patients had incomplete clinical records. A total of 136 patients were screened by diagnosis and exclusion criteria. A total of 58 septic patients developed AKI during hospitalization. The diagnosis and grading of AKI were classified according to the guidance from KDIGO Clinical Practice Guidelines for Acute Kidney Injury and Acute Kidney Injury Network (AKIN) classification [12,13]. Venous blood was drawn from all patients within 24 h of admission, and serum was collected by centrifugation and stored at −80 °C for future use. Each patient signed written informed consent, and the experimental procedure was in accordance with the guidance of the Shengli Oilfield Central Hospital Ethics Committee (Approval No. 017056).

### Cell viability assay

The cell viability was analyzed using a Cell Counting Kit-8 assay (CCK-8). HK-2 cells with a cell density of 5 × 10^3^ cells/well were seeded into 96-well cell culture plates and cultured in a cell incubator with 5% CO_2_ at 37 °C. After 48 h of incubation, the cell viability was measured by 10 μL CCK-8 regent (Bioss, Beijing, China), followed by further 1.5 h of incubation at 37 °C. Finally, the cell viability was evaluated by reading the OD value at 450 nm by a microplate reader (Bio-Rad, USA).

### Evaluation of kidney injury

To assess renal injury, we performed experiments to detect the expression levels of Scr, Cys-C and KIM-1 in the serum of patients. Serum Scr and Cys-C levels were detected by an automatic biochemical analyzer (AU400, Olympus, Tokyo, Japan), and KIM-1 levels were detected using ELISA kits (Boster Biological Technology, Wuhan, China).

### RNA extraction and quantitative real-time PCR (qRT-PCR)

The total RNA was extracted from the serum and HK-2 cells using TRIzol (Invitrogen Thermo Fisher Scientific, Inc.) as per the standard method. And the RNA were reverse-transcribed into cDNA using the PrimeScript RT reagent kit (Takara Bio Inc., Shiga, Japan). The cDNA was subsequently used as the template for qPCR, which was carried out to evaluate the expression levels of miR-574-5p using a SYBR-Green I Master Mix kit (Invitrogen; Thermo Fisher Scientific, Inc.) and the 7500 Real‑Time PCR System (Applied Biosystems, USA). U6 was used as an endogenous control and the final relative miR-574-5p expression was calculated using the 2^−ΔΔCt^ method.

### Statistical analysis

All statistical analyses used SPSS 21.0 software (IBM, Chicago, IL) and GraphPad Prism 7.0 software (GraphPad Software, Inc., USA), data were expressed as mean ± SD. The differences between groups were assessed using Student's *t*-test, Chi-square test and one-way ANOVA with Tukey's multiple comparison test. Pearson correlation coefficients were used for correlation analysis. Logistic regression analysis assessed the ability of miR-574-5p to predict the occurrence of AKI, in which the clinical data, miR-574-5p, and other factors that might be correlated with AKI occurrence were included in the analysis [14]. To facilitate the logistic analysis, all the factors analyzed were included as categorical variables. In addition to the original categorical variables, including gender, hypertension, diabetes mellitus and cardiovascular disease, the continuous variables, including age, C-reactive protein (CRP), procalcitionin (PCT), whole blood cell (WBC), estimated glomerular filtration (eGRF), Acute Physiology and Chronic Health Evaluation (APACHE) II score, Sepsis-related Organ Failure Assessment (SOFA) score, Scr, Cys-C, KIM-1 and miR-574-5p, were converted to categorical variables using median as the cutoff values. Body mass index (BMI) was divided into two groups based on a cutoff value of 24 kg/m^2^. The diagnostic value of serum miR-574-5p was evaluated by plotting a receiver operating characteristic (ROC) curve. The significance level was set at *p* < 0.05.

## Result

### MiR-574-5p promotes HK-2 cell viability

**Figure 1.**
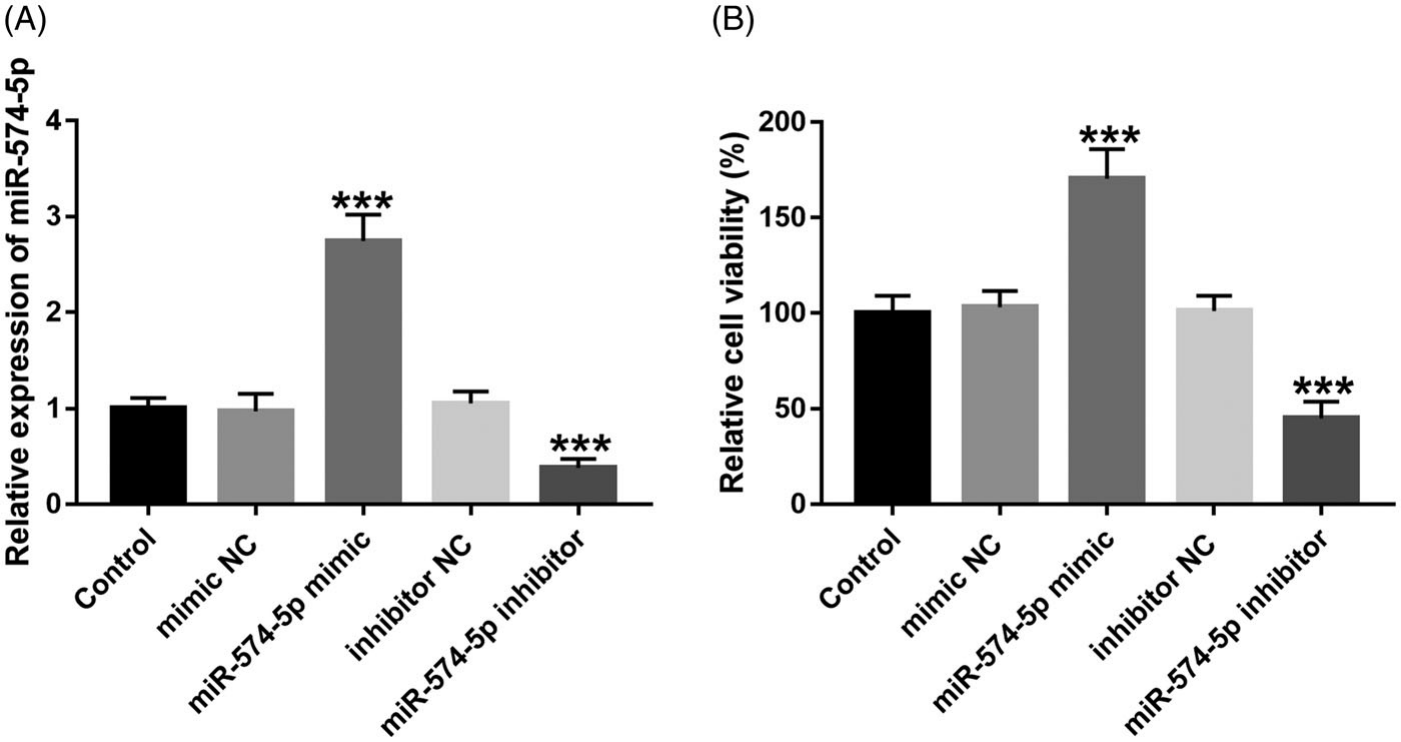
miR-574-5p promotes HK-2 cell viability. (A) After transfection with mimics of miR-574-5p, the expression level of miR-574-5p increased, while after transfection with inhibitors of miR-574-5p, the expression level of miR-574-5p decreased. (B) Overexpression of miR-574-5p can promote HK-2 cell viability, while knockdown of miR-574-5p inhibits cell viability. ***p < 0.001.

The current study conducted experiments on cells to confirm the functional role of miR-574-5p in the progression of AKI. After stable transfection with a mimic or an inhibitor, miR-574-5p expression was overexpressed or knocked down in HK-2 cell respectively, compared with the corresponding negative controls (all *p* < 0.001 Figure 1(A)). The cell viability assay revealed that overexpression of miR-574-5p contributed to increase the viability of HK-2 cells, while the inhibition of the expression of miR-574-5p inhibited the viability of HK-2 cells (all *p* < 0.001 Figure 1(B)).

### Baseline characteristics of the sepsis patients

From Table 1, there were no differences in age, BMI, gender, hypertension, diabetes mellitus, cardiovascular disease and WBC numbers between non-AKI patients and AKI patients (all *p* > 0.05). Compared with the non-AKI cases, the patients with septic AKI had significantly high levels of CRP and PCT (both *p* < 0.01). AKI patients had higher APACHE II and SOFA scores than the patients without AKI (both *p* < 0.01). In terms of the kidney injury biomarkers, the levels of Scr, Cys-C and KIM-1 were markedly higher in AKI patients than that in the non-AKI patients (all *p* < 0.001).

**Table 1.** Baseline characteristics of the enrolled sepsis patients.

Characteristics	Non-AKI (*n* = 78)	AKI (*n* = 58)	*p* Value
Age (years)	49.70 ± 15.20	54 ± 15.86	0.072
BMI (kg/m^2^)	21.28 ± 2.68	21.85 ± 2.47	0.203
Gender, males (%)	57 (73.0)	45 (77.5)	0.548
Hypertension (%)	16 (20.5)	9 (15.5)	0.457
Diabetes mellitus (%)	8 (10.2)	8 (13.7)	0.527
Cardiovascular disease (%)	4 (0.51)	3 (0.51)	0.991
CRP (ng/mL)	63.89 ± 28.33	79.55 ± 27.69	0.002
PCT (ng/mL)	3.61 ± 1.31	3.97 ± 1.48	0.002
WBC (×10^9^/L)	13.33 ± 8.09	15.19 ± 8.82	0.206
eGRF (mL/min per 1.73 m^2^)	59.11 ± 21.33	49.41 ± 19.06	0.007
APACHE II score	13.21 ± 3.97	16.14 ± 6.01	0.001
SOFA score	7.83 ± 2.86	9.72 ± 5.04	0.002
Scr (μM)	91.74 ± 33.19	156.15 ± 26.14	<0.001
Cys-C (mg/L)	0.58 ± 0.19	1.95 ± 0.57	<0.001
KIM-1 (ng/mL)	4.58 ± 0.57	22.69 ± 7.29	<0.001

BMI: body mass index; CRP: C-reaction protein; PCT: procalcitionin; WBC: white blood cell; eGRF: estimated glomerular filtration; APACHE: Acute Physiology and Chronic Health Evaluation; SOFA: Sepsis-related Organ Failure Assessment; Scr: serum creatinine; Cys-C: cystatin-C; KIM-1: kidney injury molecule-1.

### Expression of serum miR-574-5p in sepsis-induced AKI patients

**Figure 2.**
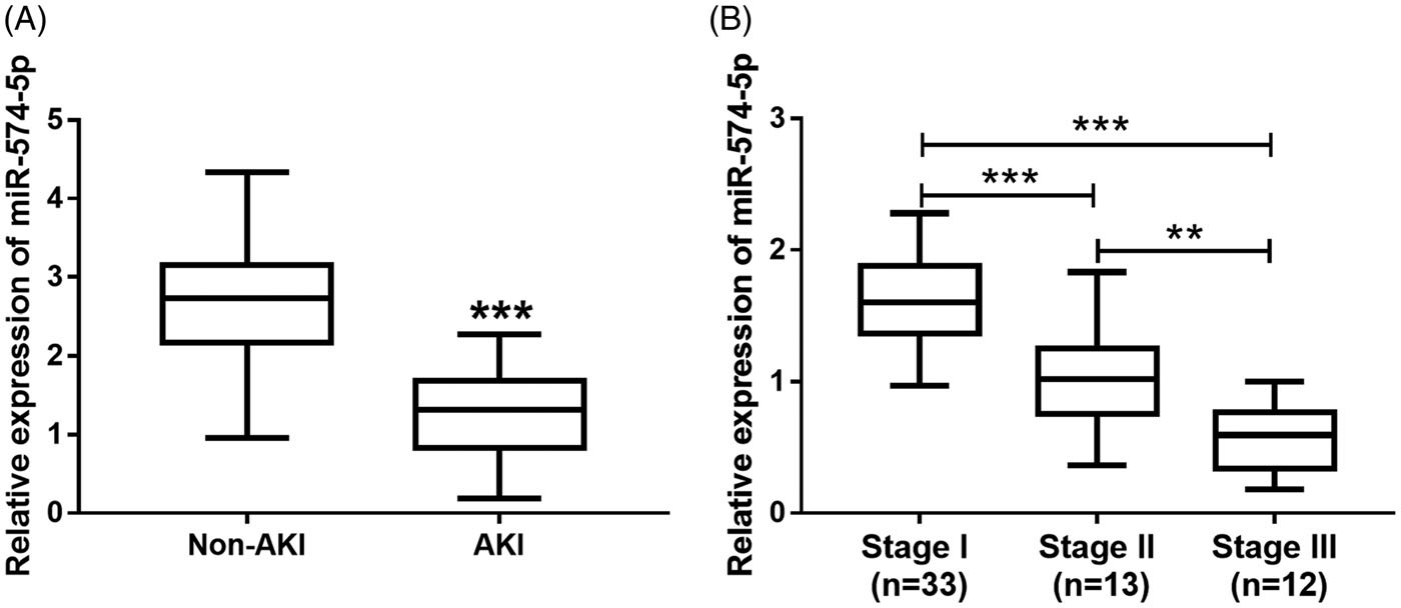
Expression of serum miR-574-5p in sepsis-induced AKI patients. (A) The expression level of miR-574-5p in AKI patients was significantly lower than that in patients without AKI. (B) MiR-574-5p expression level decreased with the increase of AKI stage. **p < 0.01 and ***p < 0.001.

The relative expression levels of miR-574-5p in AKI patients and non-AKI patients were determined by qRT-PCR. The expression level of miR-574-5p in AKI patients was significantly lower than that in patients with non-AKI (all *p* < 0.001 Figure 2(A)). AKI is classified into three levels according to the guidance from KDIGO Clinical Practice Guidelines for AKI and Acute Kidney Injury Network (AKIN) classification [12]. After AKI patients were graded, there were 33 stage I, 13 stage II and 12 stage III patients in this study. The expression level of miR-574-5p was the highest in stage I patients and the lowest in stage III patients. Therefore, the expression level of miR-574-5p decreased with the increase of AKI stage (all *p* < 0.01 Figure 2(B)).

### A negative correlation between serum miR-574-5p and kidney injury biomarkers

The correlation between miR-574-5p and Scr, Cys-C, KIM-1 was studied by Pearson correlation analysis. As shown in Table 2, the analysis results showed that miR-574-5p was significantly negatively correlated with Scr (*r* = −0.757, *p* < 0.001), Cys-C(*r* = −0.723, *p* < 0.001), KIM-1(*r* = −0.694, *p* < 0.001).

**Table 2.** Correlation between serum miR-574-5p levels and kidney injury biomarkers in AKI.

Kidney injury biomarkers	miR-574-5p	
	*r* Value	*p* Value
Scr	−0.757	<0.001
CysC	−0.723	<0.001
KIM-1	−0.694	<0.001

Scr: serum creatinine; Cys-C: cystatin C; KIM-1: kidney injury molecule-1.

### Serum miR-574-5p predicts AKI onset in patients with sepsis

To analyze the risk factors that were related with the occurrence of AKI, the clinical data and serum miR-574-5p levels were evaluated using a multiple logistic regression model. The results showed that miR-574-5p (HR = 2.852, 95% CI = 1.904–3.741, *p* = 0.012) were independently associated with the occurrence of AKI in sepsis patients, and may be a potential risk factor for the occurrence of AKI in patients with sepsis (Table 3).

**Table 3.** Risk factor analysis to predict septic AKI using multiple logistic regression model.

Indicators	Multivariate analysis		
	OR	95% CI	*p*
Age	1.598	0.899–2.367	0.271
BMI	1.633	0.879–2.744	0.200
Gender, males	1.219	0.847–1.896	0.548
Hypertension	1.222	0.851–2.642	0.457
Diabetes mellitus	1.268	0.860–1.786	0.527
Cardiovascular disease	1.396	0.785–1.946	0.891
CRP	1.618	0.910–2.854	0.202
PCT	1.696	0.908–2.385	0.140
WBC	1.598	0.896–2.964	0.203
eGRF	1.745	1.388–2.249	0.047
APACHE II score	1.845	0.945–2.845	0.071
SOFA score	1.764	0.926–2.612	0.096
Scr	2.145	1.845–3.512	0.031
Cys-C	2.018	1.820–3.336	0.034
KIM-1	1.854	1.406–2.584	0.043
miR-574-5p	2.852	1.904–3.741	0.012

BMI: body mass index; CRP: C-reaction protein; PCT: procalcitionin; WBC: white blood cell; eGRF: estimated glomerular filtration; APACHE: Acute Physiology and Chronic Health Evaluation; SOFA: Sepsis-related Organ Failure Assessment; Scr: serum creatinine; Cys-C: cystatin-C; KIM-1: kidney injury molecule-1.

### Diagnostic accuracy of serum miR-574-5p to screen AKI from sepsis patients

**Figure 3.**
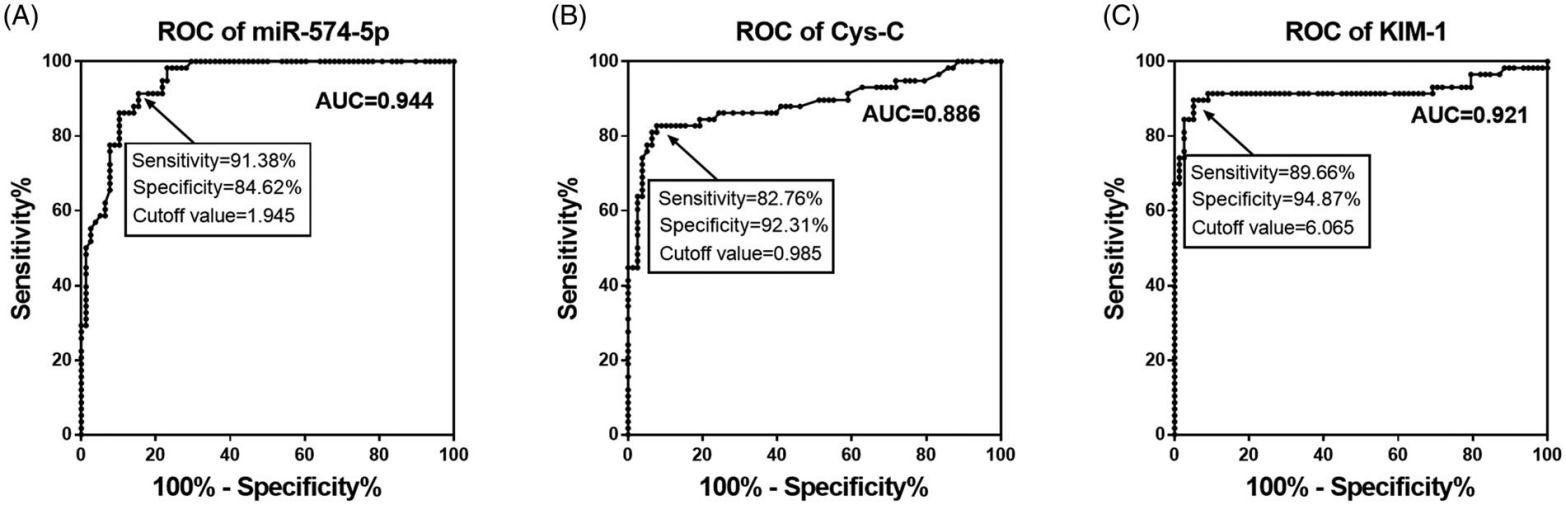
Diagnostic accuracy of serum miR-574-5p, Cys-C and KIM-1 to screen AKI from sepsis patients. (A) A receiver operating characteristic (ROC) curve based on miR-574-5p expression indicated high diagnostic accuracy of miR-574-5p. (B) A ROC curve based on Cys-C for sepsis patients. (C) A ROC curve based on KIM-1 for sepsis patients.

Because of the abnormal expression level of miR-574-5p in AKI samples, we evaluated its diagnosis significance in AKI patients. This study estimated the relationship between the expression level of miR-574-5p and AKI diagnosis by plotting ROC curve (Figure 3(A)). The results showed that miR-574-5p had a high diagnostic value with an area under the curve of 0.944, and the cutoff value was 1.945, the sensitivity was 91.38% and the specificity was 84.62%. In addition, the ROC curves of Cys-C and KIM-1 were also plotted, and the AUC was 0.886 for Cys-C and 0.921 for KIM-1 (Figure 3(B,C)).

## Discussion

Sepsis is a life-threatening clinical syndrome characterized by organ dysfunction due to the patient's dysregulated response to infection [15]. Sepsis is the most common cause of death in intensive care units (ICUs), accounting for approximately 250 000 deaths annually in the States [16]. In China, the number of sepsis-related deaths in 2015 was approximately 1 000 000 [17]. AKI caused by sepsis is one of the most common complications of sepsis. In addition, sepsis is a known cause of AKI, which increases the morbidity of patients and leads to multiple organ dysfunction [18]. Sepsis-induced AKI is particularly common in hospitalized and ICU patients, with an incidence of 11–67% and a mortality rate of 13–36% [19,20] Although we have improved our ability to treat AKI, the diagnosis and detection of AKI remains an important issue and clinical burden [3]. At present, Scr, Cys-C, KIM-1 are three important indicators for predicting acute kidney injury, and the accuracy of these molecular markers in the diagnosis of AKI is limited [21–23]. Thus new AKI biomarkers also need to be identified. In addition, recent studies have reported the potential role of microRNAs (miRNAs) as biomarkers in the diagnosis, prevention and prognosis of sepsis-induced AKI [24]. In this study, we found that miR-574-5p was down-regulated in sepsis patients with AKI, and its expression level decreased with the increase of AKI severity. This suggests that the up-regulated expression of miR-574-5p may protect against AKI. Therefore, we speculated that the decreased expression level of miR-574-5p might be a risk factor for the occurrence of AKI in sepsis patients. In order to verify our conjecture, further studies will be needed to elucidate the relationship between miR-574-5p and AKI.

Tubular epithelial cells refer to the outer layer of cells in the renal tubules [25], and increasing evidence suggests that renal tubular epithelial cell apoptosis plays an important role in sepsis-induced AKI [26,27]. Therefore, inhibiting apoptosis of renal tubular epithelial cells may be an effective strategy to prevent or treat sepsis-induced AKI. Many studies have shown that miR-574-5p plays an important role in regulating various cellular biological functions. For example, the signaling pathway PTCSC3-miR-574-5p-SCAI-Wnt/catenin mediates the proliferation and migration of PTC-1 cells, which is essential for further treatment and prognosis of papillary thyroid carcinoma [28]. MiR-574-5p affects the cell cycle distribution and apoptosis of thyroid cancer cells through catenin/Wnt signaling pathway by targeting Quaking proteins (QKIs) [29]. However, the effect of miR-574-5p on renal tubular epithelial cell is unclear. In this study, we observed that overexpression of miR-574-5p promoted the viability of HK-2 cells, while silencing of miR-574-5p inhibited cell viability. The regulatory effects of miR-574-5p on renal epithelial cells may provide novel insight into the functional role of miR-574-5p in renal cell injury-related diseases.

A previous study by Wang et al. has reported the relationship between miR-574-5p and the survival of sepsis patients, in which the low level expression of serum miR-574-5p was associated with increased morbidity, and the combination of miR-574-5p, SOFA score and sepsis stage provided a good strategy for sepsis prognosis [10]. Therefore, we speculate that miR-574-5p might play an important role in the progression of sepsis. It is well known that the development of AKI significantly increases the risk of in-hospital death in patients with sepsis [30]. Thus, the role of miR-574-5p in the occurrence and development of AKI was explored in the present study. According to the analysis results, serum miR-574-5p expression was decreased in sepsis patients who developed AKI compared with those patients without AKI. However, the expression data in healthy controls was lack in our study, owing to that healthy samples were not collected. In a previous publication by Wang et al. [10], the expression of miR-574-5p showed elevated in sepsis patients compared to healthy controls, which indicated that miR-574-5p might increase by sepsis as a protective mechanism, and increase to a lesser degree in septic patients with high severity. Thus, a significant decrease in miR-574-5p expression was observed in septic AKI cases. In addition, the Pearson correlation analysis of this study showed that the expression level of miR-574-5p was negatively correlated with renal injury markers, including Scr, Cys-C and KIM-1. Further multivariate logistic regression analysis revealed that miR-574-5p served as a risk factor for the prediction of AKI onset in sepsis patients. In addition, the ROC analysis results indicated that miR-574-5p had considerable diagnostic accuracy in septic AKI patients, suggesting that miR-574-5p may be a biomarker to predict AKI development in sepsis patients.

However, the study of miR-574-5p in this study is not thorough enough. We did not explore the specific mechanism of miR-574-5p in the pathogenesis of AKI. In addition, this study also has some defects, such as not including healthy control and lack of *in vivo* experiment. Therefore, it is hoped that more comprehensive samples will be included in future studies for confirmation analysis and further exploration of the role of miR-574-5p in the occurrence and development of AKI.

In conclusion, this study firstly provides evidence for the regulatory effects of miR-574-5p on HK-2 cell viability. The data show that the expression level of miR-574-5p is decreased in septic AKI patients, and is associated with disease severity and kidney injury maker levels. In addition, the down-regulated expression of miR-574-5p may be used as a risk factor for predicting the occurrence of AKI in sepsis patients, with considerable accuracy in AKI screening.

## Data Availability

All data generated or analyzed during this study are included in this published article.
